# Predictive Modeling of Riboflavin Production in *Lactiplantibacillus plantarum* MTCC 25432 Using Fuzzy Inference System

**DOI:** 10.3390/foods12173155

**Published:** 2023-08-22

**Authors:** Vikram Kumar, Vinkel Kumar Arora, Ananya Rana, Ankur Kumar, Neetu Kumra Taneja, Jayesh J. Ahire

**Affiliations:** 1 Department of Basic and Applied Sciences, National Institute of Food Technology Entrepreneurship and Management (NIFTEM), HSIIDC, Kundli, Sonipat 131028, Haryana, India; vikramkr.7496@gmail.com (V.K.); vinkelarora17@gmail.com (V.K.A.); ananya.rana.333@gmail.com (A.R.); ankur_chem97@rediffmail.com (A.K.); 2 Centre for Advanced Translational Research in Food Nanobiotechnology (CATR-FNB), National Institute of Food Technology Entrepreneurship and Management (NIFTEM), Sonipat 131028, Haryana, India; 3 Dr. Reddy’s Laboratories Limited, Hyderabad 500016, Telangana, India

**Keywords:** *Lactiplantibacillus plantarum* MTCC 25432, riboflavin, casamino acid

## Abstract

Riboflavin (Vitamin B_2_) is an essential vitamin and a microbial metabolite produced by some lactic acid bacteria (LAB). This investigation aims to study the overproduction of riboflavin in selected *Lactiplantibacillus plantarum* strain by using the one factor at a time (OFAT) tool coupled with the Fuzzy Inference System (FIS) and its validation through fermentative production in semi-defined media. Out of three *Lactiplantibacillus* strains used in this study, the maximum riboflavin producing strain was selected based on its ability to grow and produce higher levels of riboflavin. In results, *Lactiplantibacillus plantarum* strain MTCC 25432 was able to produce 346 µg/L riboflavin in riboflavin deficient assay medium and was investigated further. By using the OFAT–fuzzy FIS system, casamino acid in the range of 5–20 g/L, GTP 0.01–0.04 g/L, sodium acetate 5–15 g/L, and glycine 5–15 g/L were used to predict their effect on riboflavin production. The conditions optimized with modeling showed a 24% increment in riboflavin production (429 µg/L) by *Lactiplantibacillus plantarum* MTCC 25432 vis-a-vis the unoptimized counterpart (346 µg/L). In conclusion, an FIS-based predictive model was effectively implemented to estimate the riboflavin within an acceptable limit of 3.4%. Riboflavin production enhancing effects observed with various levels of sodium acetate, casamino acid, and GTP could be useful to re-design matrices for riboflavin production.

## 1. Introduction

Riboflavin, or vitamin B_2_, is a water-soluble vitamin crucial for optimal body development, the formation of red blood cells, and the metabolism of carbohydrates and fatty acids [1]. Riboflavin is a precursor molecule for flavozymes, flavin mononucleotide (FMN), and flavin adenine dinucleotide (FAD), which act as coenzymes in various oxidation-reduction reactions in biological systems [2]. Riboflavin must be consumed as part of the human diet, as it cannot be synthesized by humans. The Food and Nutrition Board of the USA recommends a typical adult’s daily intake of riboflavin be 1.3 mg [3], as it cannot be stored in the body and excess consumption is excreted in urine [4]. Both developing and developed nations frequently experience subclinical riboflavin deficiency [5]. Many nations, including the USA, Canada, and Argentina, have implemented obligatory fortification programs with various vitamins and minerals that contain riboflavin in their composition to address this issue [6]. On an industrial scale, riboflavin can be produced via biological or chemical techniques. In contrast to the latter, biological processes are employed more frequently due to reduced cost, waste, and energy. Furthermore, biologically derived riboflavin meets the customers increased demand for ‘natural’ food ingredients without added chemicals. Thus, microbial fermentation is a more sustainable and practical substitute for fortification, which might raise vitamin content in food products and create an imbalance [7].

At present, the utilization of lactic acid bacteria (LAB) for the production of B-group vitamins, including riboflavin, folate, and vitamin B_12_, offers an alluring method for obtaining fermented bio-enriched foods [8]. Some LAB possess the ability to produce higher concentrations of riboflavin, which may be harnessed through metabolic engineering and/or exposure to different medium constituents such as guanosine triphosphate (GTP), casamino acid, purine analogues, and chemical mutagen roseoflavin. Specifically, several strains of *Lactiplantibacillus plantarum*, *Limosilactobacillus fermentum*, *Propionibacterium*, and *Lactococcus lactis* are used for the biofortification of different vitamin-enriched foods with modifications in a chemically defined medium [5]. Capozzi and co-workers [9] reported a higher amount of riboflavin production by *Lactiplantibacillus plantarum* CRL725 in semi-defined medium (SDM). Additionally, a chemically defined medium is a requirement for physiological investigations since it enables testing in controlled conditions with access to all nutrient sources. The molecular mechanism that governs the synthesis of riboflavin is controlled by the FMN riboswitch that regulates the riboflavin biosynthesis genes *ribH*, *ribA*, *ribB*, *ribC*, and *ribG* [10].

It is often difficult to ensure invariability and increased efficacy in a complicated biological system like fermentation. The best experimental design can be difficult to achieve, especially when dealing with multilevel components [11]. In addition to the well-known and flexible one factor at a time (OFAT) approach, generalized subset designs (GSDs) are recommended as a potent tool to alter the experimental combinations. Additionally, the Fuzzy Inference System (FIS), a hybrid learning tool (Artificial Neural Network) and robust system (Fuzzy Logic), can be used to predict the efficacy of statistical experimental design, the maximum level of production of a particular biomolecule such as riboflavin, vitamin B_12_, and folate, and the relationship between fermentation variables [12].

The purpose of this study was to screen the riboflavin-producing strains *Lactiplantibacillus plantarum* MTCC 25432, *L. plantarum* MTCC 25433, and *L. plantarum* MTCC 25434 in riboflavin-free assay medium for overproduction thereof. In addition, the highest riboflavin-producing strain was investigated for optimum production of riboflavin in riboflavin-free SDM and different growth conditions using a traditional experimental design tool (OFAT) coupled with a Fuzzy Inference System (FIS) with a statistical prediction of the final production of riboflavin by using fermentation variables.

## 2. Materials and Methods

### 2.1. Bacterial Strains, Media, and Growth Conditions 

Riboflavin-producing *Lactiplantibacillus plantarum* strains *viz*., MTCC 25432 (BBC 32B), MTCC 25433 (BBC 33), and MTCC 25434 (BIF 43), were previously isolated by our research group at Microbiology Laboratory, National Institute of Food Technology Entrepreneurship and Management-Kundli (NIFTEM-K), India [13]. Prior to the experiments, the cultures were revived, checked for purity, and stored at −20 °C in 10% (*w*/*v*) reconstituted skim milk containing 0.5% (*w*/*v*) yeast extract, 1.0% (*w*/*v*) glucose, and 10% (*v*/*v*) glycerol. The highest riboflavin-producing strain was selected based on strains’ ability to grow in riboflavin-deficient media and riboflavin production thereof [13].

Commercially available riboflavin-deficient (Riboflavin Assay Medium (RAM), Difco, Sparks, MD, USA) and semi defined media (SDM, in house) were used for adaptation related studies as described earlier [14]. The composition of SDM is shown in Table 1. The components, which are heat labile, were filter sterilized (0.2 µm, cellulose acetate, Sartorius, Arvada, CO, USA) separately and stored at 4 °C. Cysteine solution was prepared fresh and added before the use. All media ingredients were of analytical grade (HiMedia, Mumbai, India).

A 1.0% (*v*/*v*) actively growing cells of selected bacteria were transferred into 10 mL fresh MRS (De Man, Rogosa and Sharpe) broth (HiMedia, Mumbai, India) and incubated at 37 °C for 16–18 h. After incubation, the cells were harvested by centrifugation at 8000× *g* for 10 min, washed twice with PBS buffer (pH 7.3), and re-suspended in same. The cell suspension (1.4 × 10^8^ CFU/mL) obtained using the above process was used to inoculate SDM with initial optical density of 0.6 units at 600 nm as determined using a UV spectrophotometer (Analytik Jena, Delhi, India).

### 2.2. Impact of Different Concentrations of Selected SDM Ingredients on the Production of Riboflavin

Different concentrations of sodium acetate (5, 10, 15 g/L), casamino acid (5, 10, 20 g/L), guanosine triphosphate (0.01, 0.02, 0.04 g/L), and glycine (5, 10, 15 g/L) were added to SDM and their impact on riboflavin production was investigated. The SDM without culture served as negative growth control.

### 2.3. Riboflavin Extraction 

Riboflavin was extracted from the samples using a method described by Juarez del Valle et al. [14] with slight modifications. In short, a 10 mL sample was mixed equally with 1.0% (*v*/*v*) acetic acid and autoclaved at 121 °C for 20 min. The mixture was cool at room temperature and centrifuged at 8000× *g* for 20 min. The supernatant was collected and filtered using 0.22 µm nylon-66 membrane filter (Nupore, Ghaziabad, India). The samples were kept at −20 °C until the riboflavin quantification.

### 2.4. Riboflavin Quantification 

Riboflavin quantification was performed using Waters HPLC (model 2707, Waters, Kolkata, India) equipped with reverse-phase C_18_ column (Kromasil, Sigma, St. Louis, MO, USA, 5 µ 100 Å, 250 × 4.6 mm), in-line degasser, auto-sampler, binary pump control (module II), and fluorescence detector (excitation wavelength: 440 nm and emission wavelength: 520 nm). All samples were analyzed using isocratic elution in freshly prepared mobile phase of methanol/water 35:65 (*v*/*v*) with flow rate of 1 mL/min. A pure form of riboflavin (Sigma, St. Louis, MO, USA) served as a control.

### 2.5. One Factor at a Time 

To obtain the highest value of vitamin B_2_ using *L. plantarum* MTCC 25432 via traditional fermentation method, a one factor at a time (OFAT) and Fuzzy Inference System (FIS) were used. In the OFAT method, various medium constituents (casamino acid 2–20 g/L, sodium acetate 5–15 g/L, GTP 0.01–0.04 g/L, glycine 5–15 g/L) were used at the level of different concentrations as described previously [14]. OFAT approach is a single factor approach used to investigate the effect of individual parameters on the fermentation process within the specified range. This method is based on the conventional approach of modifying one independent variable at a time while maintaining the other independent variables at a fixed level. It offers the opportunity to optimize medium components as well as process conditions. This method has the benefit of being straightforward, simple, and making it easy to visualize the individual effects of medium components and process conditions on a graph. However, this method has some drawbacks, such as ignoring interactions between the components, being time-consuming and expensive for many variables due to the number of experiments. It is the most often used way for enhancing the fermentation medium and process conditions.

### 2.6. Predictive Modeling Using Fuzzy Inference (FIS) Approach

A computationally sophisticated technique called a Fuzzy Inference System (FIS) computes outputs depending on current inputs and fuzzy inference rules. These methods are grounded in fuzzy logic [15]. Fuzzification, inference, and defuzzification techniques are used in FIS methodology. Fuzzification is the process of converting provided inputs into fuzzy inputs by mapping the inputs to fuzzy sets established in the relevant universe [16]. The decision-making inference process fuzzy inference rules generate the associated fuzzy outputs from these inputs, while the results of defuzzification generates nonfuzzy [17] outputs. In order to get the maximum riboflavin production, the traditional (OFAT) and an evolutionary hybrid (FIS) algorithms were used. The MATLAB 17.0 fuzzy logic toolbox was used for the FIS algorithm. The input variable was prerequisite and merges with Mamdani Fuzzy Inference System for predictive modeling in FIS.

**Figure 1:**
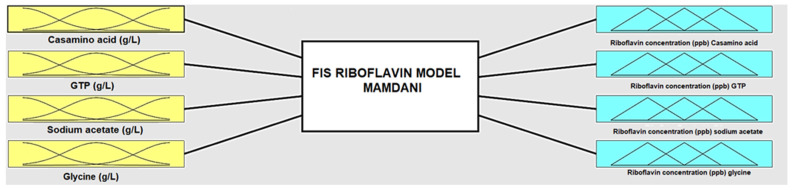
Structure of Fuzzy Inference System.

The Fuzzy Inference System (FIS) system was used to predict the effect of the parameters i.e., casamino acid 5–20 g/L, GTP 0.01–0.04 g/L, sodium acetate 5–15 g/L, and glycine 5–15 g/L on the production of riboflavin. The FIS model structure created for this study is shown in Figure 1. The Mamdani fuzzy inference system is adopted for predictive modeling in FIS. The one factor at a time was considered to investigate the effect of parameters on the production of riboflavin. The experiments were numbered from 0 to 3 i.e., 0 for the first factor and 3 for the last factor for the predictive FIS model.

**Figure 2:**
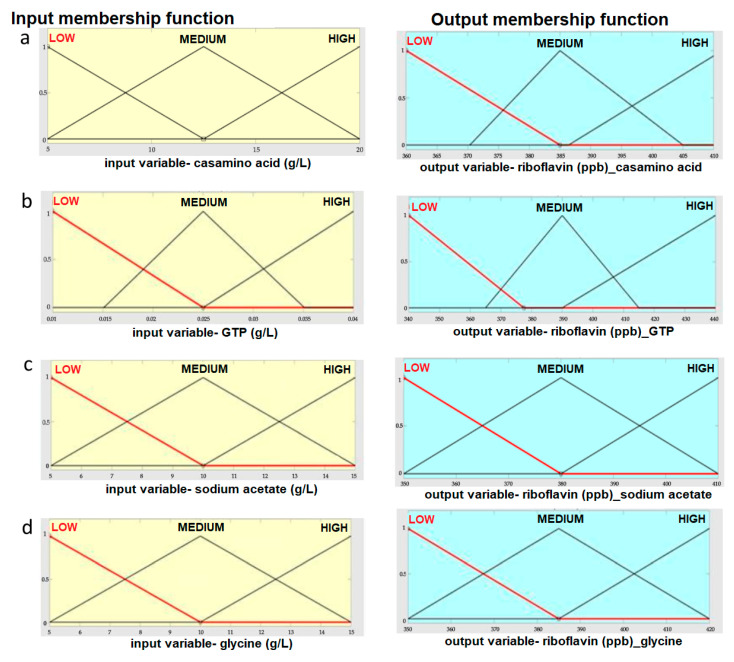
The input and output membership function of FIS model (**a**) casamino acids (g/L); (**b**) GTP (g/L); (**c**) sodium acetate (g/L); (**d**) glycine (g/L).

For the input parameter of casamino, the triangular membership function was used with the range of 5–20 g/L. The low, medium, and high peaks were at 5, 12.5, and 20 g/L. The output riboflavin concentration was also considered as triangular membership function as depicted in Figure 2a. The second parameter GTP was also modeled using triangular membership function with the range 0.01–0.04 g/L. For input parameter sodium acetate, the triangular membership function was used with the range 5–15 g/L. The low, medium, and high peaks were at 5, 10, and 15 g/L. Similarly, for glycine, the triangular membership function was used with the range 5–15 g/L. The low, medium, and high peaks were at 5, 10, and 15 g/L. The output parameter, i.e., riboflavin, was modeled using a triangular membership function range of 340–440 ppb. The input and output membership functions of the FIS model are represented in Figure 2. 

### 2.7. Statistical Analysis 

The obtained data from the separate experiments were analyzed and expressed in mean ± SD. For tabulation, descriptive visualization, and analysis of variance (ANOVA), Microsoft Office (2007) was used among the values of statistical significance. The prediction and validation-related FIS work was done using the fuzzy logic toolbox of MATLAB 17.0. The MODDE 12.0 software was used to obtain prediction-based experimental designs in this research.

## 3. Results and Discussion 

### 3.1. Selection of Riboflavin Overproducing Strain

**Figure 3:**
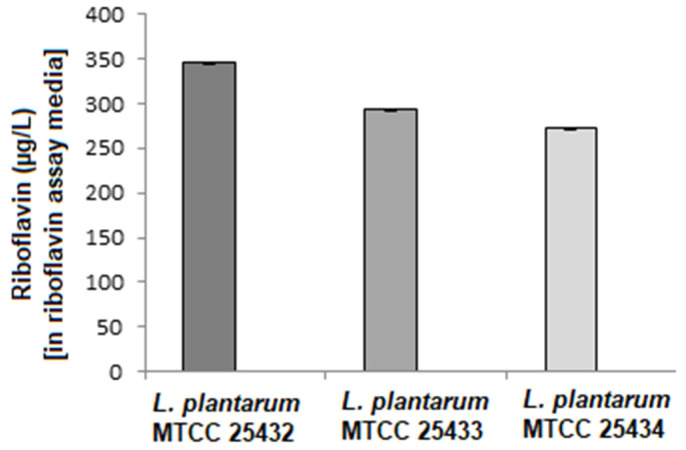
Riboflavin production of *L. plantarum* MTCC 25432, MTCC 25433, and MTCC 25434.

Among the three strains of *Lactiplantibacillus plantarum* MTCC 25432, MTCC 25433, and MTCC 25434, the *L. plantarum* MTCC 25432 was able to overproduce riboflavin as compared to rest of the strains (Figure 3). These results coordinated well with our previous findings [13,18,19]. The selected strain was used to optimize overproduction of riboflavin using OFAT-FIS model.

### 3.2. Impact of Different Concentrations of Some Selected SDM Nutrient, Ingredients on the Production of Riboflavin 

Bacterial cell growth and riboflavin production were both impacted by the presence of casamino acid, a compound made up of a combination of amino acids and short peptides obtained from the acid hydrolysis of casein [14]. As a result, adding 20 g/L of casamino acid to SDM increased riboflavin production by around 16% (*v*/*v*), whereas adding 25–30 g/L of casamino acid to this medium had no effect on riboflavin production. The amount of riboflavin production increased noticeably up to 24% (*v*/*v*) when SDM medium was supplemented with 0.04 g/L of GTP, one of the building blocks for de novo riboflavin biosynthesis. The bacterial growth and vitamin synthesis were affected by adding the glycine 15 g/L but when added up to 20 g/L, the production of riboflavin further decreased. The presence of the GTP 0.04 g/L highly increased the production of riboflavin up to 24% (*v*/*v*) (Table 2). These results indicate dose-dependent action of media components, which may influence the production of riboflavin by acting as a direct precursor of riboflavin. Juarez del Valle et al. [14] showed a significant correlation between the expansion of the bacterial growth in the SDM and synthesis of the riboflavin by the *L. plantarum* CRL 725. 

In this study, the presence of the GTP (0.4 g/L) in the SDM showed higher riboflavin production. Furthermore, GTP (0.01, 0.02, 0.04 g/L) and casamino acid (5–20 g/L) in SDM provided the best growth conditions to *L. plantarum* MTCC 25432. As studied earlier by Jaurez and colleague, the absence of the GTP in the riboflavin free assay medium, low riboflavin production is reported in the *L. plantarum* CRL 725 [14], but the effect on the growth of bacteria was not reported. However, the higher concentration of the casamino acid (up to 30 g/L) was responsible for reducing the viability of the bacterial cells; in these circumstances, the casamino acid would function as an inhibitor for cellular growth [20].

The presence of amino acids such as asparagine and glycine in the synthetic media is known to influence the growth of some lactic acid bacteria (LAB). For instance, the asparagine is necessary for the growth of LAB from the wines; glycine is required to enhance the growth of LAB isolated from the human origin samples [21]. In addition, the removal of glycine from the growth medium affects the production of riboflavin; the mechanism is still not well defined. Additionally, casamino acid has a high concentration of free amino acids, which act as building blocks for the metabolism of purines and purine compounds, which directly act as riboflavin precursors. These findings could perhaps explain a higher level of gene expression involved in the production of riboflavin when casamino acids were introduced in the SDM [22].

Guanosine triphosphate (GTP) and ribose 5-phosphate can act as a direct precursor molecule to produce the riboflavin. Since guanosine contains phosphate that is normally eliminated during the synthesis of riboflavin, its impact on riboflavin production has been studied in *Eremothecium ashbyii* [23]. The GTP was added (0.04 g/L) in the SDM; however, it had no effect on the growth of *L. plantarum* MTCC 25432, but it improved the production of riboflavin.

### 3.3. Riboflavin Quantification 

The HPLC was used to quantify the concentration of riboflavin after the fermentation in SDM with variable components and growth conditions, as discussed earlier. The higher concentration of riboflavin obtained when enriched with sodium acetate 5–10 g/L, i.e., 429.18 ppb. As compared to the different growth conditions (time, temperature, and pH), the highest production of riboflavin was obtained at 35 °C, 12 h, and pH 6.5. As far as we know, this is the first report where the effect on biosynthesis of riboflavin in the presence of different medium components as well as different growth conditions has been evaluated independently. The improved culture and growth conditions described in this research resulted in higher levels (the highest 24% increase) of riboflavin in the strain *L. plantarum* MTCC25432.

### 3.4. Predictive Modeling Using OFAT-FIS Approach

The different medium components such as sodium acetate (5, 10, 15 g/L), casamino acid (5, 10, 20 g/L), guanosine triphosphate (GTP) (0.01, 0.02, 0.04 g/L), and glycine (5, 10, 15 g/L) were used. The results showed a significant (*p* < 0.05) increase in the production riboflavin. The greatest production level of >429 ppb was obtained when the above parameters were used sequentially to optimize fermentation by *L. plantarum* MTCC 25432.

**Figure 4:**
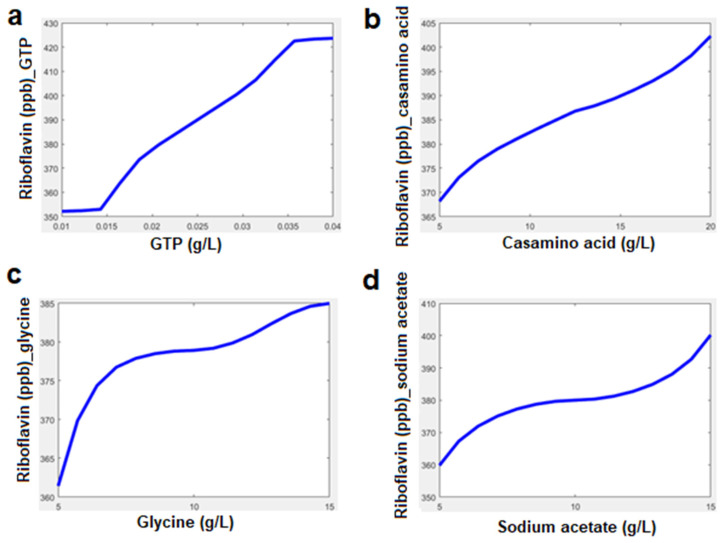
Effect of the different concentrations of GTP (**a**), casamino acid (**b**), glycine (**c**), and sodium acetate (**d**) on the production of the riboflavin.

*Lactiplantibacillus plantarum* MTCC 25432 produced 24% higher riboflavin when grown in the presence of 0.4 g/L GTP in the SDM (Figure 4a). Similarly, when adding 20 g/L casamino acid to the SDM, it increased the production of riboflavin by 17% (Figure 4b), while high concentrations of casamino acid up to 30 g/L showed the reverse effect of the production of riboflavin. In the presence of glycine, a rapid increase in the production of riboflavin was measured, but as the concentration of glycine was increased up to 15 g/L, the production of riboflavin was reversely affected (Figure 4c). Moreover, sodium acetate also showed variation in the microbial production of riboflavin. The presence of a lower concentration of sodium acetate (up to 10 g/L) influences the production of riboflavin, while at a higher concentration, riboflavin production is reduced (Figure 4d).

**Figure 5:**
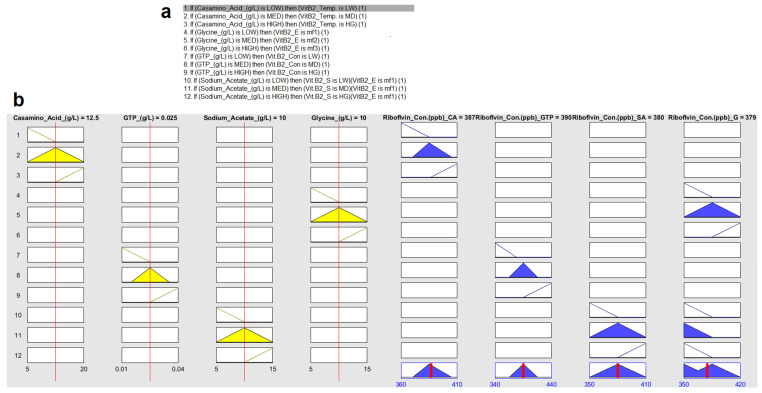
(**a**) Rules for FIS, (**b**) inputs and outputs rules for FIS model (in Δ yellow (**left**) and in Δ blue (**right**), respectively.

The FIS is a statistical method for processing data that borrows concepts from the gene theory of evolution and biological neurons. It offers a useful convergence criterion for hiding nodes, which leads to the fitting of a plausible function. Rules for FIS with inputs and outputs are depicted in Figure 5. The input and output rules for FIS are predicted at higher concentrations of the medium components and riboflavin production, respectively. The predictive model in the current investigation was extremely accurate and best fit [12], with a significant RMSE of 2.1352 and an R-value of 0.9985 for *L. plantarum* MTCC 25432. Figure 5a depicts the rules set for the Fuzzy Inference System. Figure 5b depicts the input parameters, i.e., casamino acid 5–20 g/L, GTP 0.01–0.04 g/L, sodium acetate 5–15 g/L, and glycine 5–15 g/L and output parameter, i.e., riboflavin concentration, along with their triangular membership functions.

**Figure 6:**
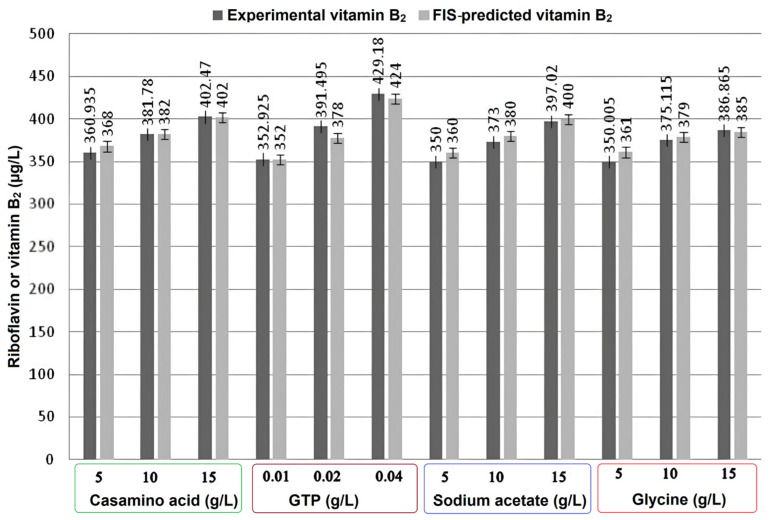
Experimental and FIS-predicted values of vitamin B_2_ (µg/L) by variation in individual parameters.

Figure 6 depicts the experimental and FIS-predicted values of vitamin B_2_ by variation in individual parameters, i.e., casamino acid 5–20 g/L, GTP 0. 01–0. 04 g/L, sodium acetate 5–15 g/L, and glycine 5–15 g/L. For 5 g/L casamino acid, the experimental value of riboflavin production was observed to be 360.93 µg/L, while the FIS predicted a value of 368 µg/L. For casamino acid, the highest variation between experimental and FIS predicted values was found to be in the range of 0.12% to −1.96%. For GTP 0.02 g/L, the experimental and FIS predicted values were 391.495 µg/L and 398 µg/L, respectively. Similarly, for the other parameters, i.e., GTP, sodium acetate, and glycine, the variation in the experimental and FIS-predicted models was found to be in the range of −0.26% to 3.4%, −0.75% to −2.8%, and 0.48% to −3.1% respectively. Hence, the FIS model can be used to efficiently predict the effect of individual parameters.

For validation of the FIS, experiments were conducted with each individual factor at a value in between the selected range, i.e., casamino acid 11 g/L, GTP 0. 03 g/L, sodium acetate 12 g/L, and glycine 12 g/L, and experimental riboflavin was determined. The FIS model was used to predict the riboflavin values for the given values of the input parameters. Table 2 shows that the experimental and predicted values for casamino acid, GTP, sodium acetate, and glycine are 386.915, 403.18, 385.14, and 380.69, and 384, 402, 382, and 383 µg/L, respectively. The variation between the results was found to be in the range of −0.61 to 0.82%. Hence, the FIS model can be used to predict riboflavin.

FIS alone and combined with GSD design has been described by other researchers as an accurate tool for the optimization, prediction, and validation of the fermentation process with R-values of 0.957 [12,24]. On an industrial, economic, and environmental scale, the improved riboflavin production process may be deemed practical due to encouraging findings during production [19,25]. For the best riboflavin-producing strain *L. plantarum*, MTCC 25432, the GTP (0.04 g/L), temperature 35 °C, time 12 h, and pH 6.0 are more favorable for the highest riboflavin production, although a sharp decrease in the production was observed at other conditions. The riboflavin production in the present study using *L. plantarum* MTCC 25432 increased around 24% (429 µg/L), which is greater than the previously reported 3.5-fold increase in *L. plantarum* CRL 725 [14].

Our findings are also supported by the previous study where ribulose-5-phosphate was used as a carbon source in the medium, which influenced the overexpression of the *ribA* gene and, as a result overproduction of riboflavin [26]. Enrichment of the medium using different carbon and nitrogen sources was more effective during metabolism, indicating that these traits would be necessary and advantageous in several ecological niches, demonstrating considerable ecological adaption to various growth conditions and growth substrates [27]. Glycine was a necessary amino acid for the growth of several lactic acid bacteria isolated from the wine and acted as a precursor molecule for several metabolites. Our research also supported some prior findings (through increased riboflavin production) about the amino acid requirements of *L. plantarum* [28].

## 4. Conclusions

At present, few strains of lactic acid bacteria have been identified that can produce riboflavin in an appropriate amount. In this study, we have determined the production of riboflavin by *Lactiplantibacillus plantarum* MTCC 25432 using an SDM with variable amounts of essential components and growth parameters. The improved growth conditions outlined in this study enabled the production of riboflavin at higher levels (almost 24%). An FIS-based predictive model was effectively implemented to estimate riboflavin within acceptable limits of 3.4%. Riboflavin production enhancing effects observed with various levels of sodium acetate, casamino acid, and GTP could be useful to re-design matrices for riboflavin production.

## Figures and Tables

**Table 1:** Chemical composition of semi-defined media (SDM).

Ingredients	Quantity (g/L)
Sodium acetate	5.0
Ammonium citrate	1.0
MgSO_4_·7H_2_O	0.4
MnSO_4_·7H_2_O	0.038
KH_2_PO_4_	3.0
K_2_HPO_4_	3.0
FeSO_2_	0.02
Tween 80	1.0
Sucrose	20.0
NaCl	0.02
l-Aspartic acid	0.020
l-Asparagine	0.60
l-Phenylalanine	0.1
l-Tyrosine	0.1
l-Glutamic acid	0.2
l-Glutamine	0.2
l-Tryptophan	0.2
l-Cysteine	0.2
Uracil	0.02
Guanosine	0.02
Adenine	0.02
Xanthine	0.02
Orotic acid	0.002
Biotin	0.01
*p*-aminobenzoate	0.01
Pantothenic acid	0.001
Nicotinic acid	0.001
Thiamine	0.001
Pyridoxal	0.004
B_12_	0.001
Folic acid	0.001

**Table 2:** Validation of FIS model.

Sr. No.	Parameter	Value	FIS-Predicted Riboflavin (µg/L)	Experimental Riboflavin (µg/L)	% Variation
1.	Casamino	11	384	386.915	0.75
2.	GTP	0.03	402	403.18	0.29
3.	Sodium acetate	12	382	385.14	0.82
4.	Glycine	12	383	380.69	−0.61

## Data Availability

Data is contained within the article.

## References

[1] Fraaije M.W., Mattevi A. (2000). Flavoenzymes: Diverse catalysts with recurrent features. Trends Biochem. Sci..

[2] Burgess C.M., Smid E.J., Rutten G., van Sinderen D. (2006). A general method for selection of riboflavin-overproducing food grade micro-organisms. Microb. Cell Factories.

[3] Schallmey M., Singh A., Ward O.P., Sumi C.D., Yang B.W., Yeo I.-C., Hahm Y.T., Zhang C., Zhang X., Yao Z. (2004). Developments in the use of *Bacillus* species for industrial production. Can. J. Microbiol..

[4] Oakley G.P., Tulchinsky T.H. (2010). folic acid and vitamin B_12_ fortification of flour: A global basic food security requirement. Public Health Rev..

[5] Thakur K., Lule V.K., Rajni C.S., Kumar N., Mandal S., Anand S., Kumari V., Tomar S.K. (2016). Riboflavin producing probiotic lactobacilli as a biotechnological strategy to obtain riboflavin-enriched fermented foods. J. Pure Appl. Microbiol..

[6] Palacios C., Hofmeyr G.J., Cormick G., Garcia-Casal M.N., Peña-Rosas J.P., Betrán A.P. (2020). Current calcium fortification experiences: A review. Ann. N. Y. Acad. Sci..

[7] LeBlanc J., Laiño J., del Valle M.J., Vannini V., van Sinderen D., Taranto M., de Valdez G.F., de Giori G.S., Sesma F. (2011). B-Group vitamin production by lactic acid bacteria—Current knowledge and potential applications. J. Appl. Microbiol..

[8] Gu Q., Li P. (2016). Biosynthesis of vitamins by probiotic bacteria. Probiotics and Prebiotics in Human Nutrition and Health.

[9] Capozzi V., Russo P., Dueñas M.T., López P., Spano G. (2012). Lactic acid bacteria producing B-group vitamins: A great potential for functional cereals products. Appl. Microbiol. Biotechnol..

[10] Mishra V., Rana A., Ahire J.J. (2022). Riboswitch-mediated regulation of riboflavin biosynthesis genes in prokaryotes. 3 Biotech.

[11] Surowiec I., Vikström L., Hector G., Johansson E., Vikström C., Trygg J. (2017). Generalized subset designs in analytical chemistry. Anal. Chem..

[12] Kumari M., Bhushan B., Kokkiligadda A., Kumar V., Behare P., Tomar S. (2021). Vitamin B_12_ biofortification of soymilk through optimized fermentation with extracellular B_12_ producing *Lactobacillus* isolates of human fecal origin. Curr. Res. Food Sci..

[13] Bhushan B., Kumkum C., Kumari M., Ahire J.J., Dicks L.M., Mishra V. (2020). Soymilk bio-enrichment by indigenously isolated riboflavin-producing strains of *Lactobacillus plantarum*. LWT.

[14] del Valle M.J., Laiño J.E., de Giori G.S., LeBlanc J.G. (2016). Factors stimulating riboflavin produced by *Lactobacillus plantarum* CRL 725 grown in a semi-defined medium. J. Basic Microbiol..

[15] El-Gamal M., Abdulghafour M. (2003). Fault isolation in analog circuits using a fuzzy inference system. Comput. Electr. Eng..

[16] Tomasiello S., Pedrycz W., Loia V. (2022). Fuzzy inference systems. Contemporary Fuzzy Logic: Big and Integrated Artificial Intelligence.

[17] Siler W., Buckley J.J. (2004). Fuzzy Expert Systems and Fuzzy Reasoning.

[18] Kumar V., Amrutha R., Ahire J.J., Taneja N.K. (2022). Techno-functional assessment of riboflavin-enriched yogurt-based fermented milk prepared by supplementing riboflavin-producing probiotic strains of *Lactiplantibacillus plantarum*. Probiotics Antimicrob. Proteins.

[19] Kumar V., Ahire J.J., Amrutha R., Nain S., Taneja N.K. (2023). Microencapsulation of riboflavin-producing *Lactiplantibacillus plantarum* MTCC 25432 and evaluation of its survival in simulated gastric and intestinal fluid. Probiotics Antimicrob. Proteins.

[20] Narisetty V., Prabhu A.A., Bommareddy R.R., Cox R., Agrawal D., Misra A., Haider M.A., Bhatnagar A., Pandey A., Kumar V. (2022). Development of hypertolerant strain of *Yarrowia lipolytica* accumulating succinic acid using high levels of acetate. ACS Sustain. Chem. Eng..

[21] Kieliszek M., Pobiega K., Piwowarek K., Kot A.M. (2021). Characteristics of the proteolytic enzymes produced by lactic acid bacteria. Molecules.

[22] Abbas C.A., Sibirny A.A. (2011). Genetic control of biosynthesis and transport of riboflavin and flavin nucleotides and construction of robust biotechnological producers. Microbiol. Mol. Biol. Rev..

[23] Mitsuda H., Nakajima K. (1975). Guanosine nucleotide precursor for flavinogenesis of *Eremothecium Ashbyii*. J. Nutr. Sci. Vitaminol..

[24] Kim S.-H., Singh D., Son S.Y., Lee S., Suh D.H., Lee N.-R., Park G.-S., Kang J., Lee C.H. (2023). Characterization and temporal dynamics of the intra- and extracellular environments of *Lactiplantibacillus plantarum* using multi-platform metabolomics. LWT.

[25] Tolar J.G., Li S., Ajo-Franklin C.M. (2023). The differing roles of flavins and quinones in extracellular electron transfer in *Lactiplantibacillus plantarum*. Appl. Environ. Microbiol..

[26] Birkenmeier M., Neumann S., Röder T. (2014). Kinetic modeling of riboflavin biosynthesis in *Bacillus subtilis* under production conditions. Biotechnol. Lett..

[27] Douillard F.P., Ribbera A., Kant R., Pietilä T.E., Järvinen H.M., Messing M., Randazzo C.L., Paulin L., Laine P., Ritari J. (2013). Comparative genomic and functional analysis of 100 *Lactobacillus rhamnosus* strains and their comparison with strain GG. PLoS Genet..

[28] Møretrø T., Hagen B.F., Axelsson L. (1998). A new, completely defined medium for meat lactobacilli. J. Appl. Microbiol..

